# B-cell extracellular vesicle biomarkers for detection of antibody-mediated rejection in lung transplantation

**DOI:** 10.1016/j.xjtc.2025.03.005

**Published:** 2025-03-18

**Authors:** Laxminarayana Korutla, Yun Zhu Bai, Nicole DeMarais, Sriharsha Talapaneni, Robert Hu, Vincent Burke, Daniel Kreisel, Prashanth Vallabhajosyula

**Affiliations:** a Department of Surgery, Yale School of Medicine, New Haven, Conn; b Department of Surgery, Washington University School of Medicine, St Louis, Mo

**Figure:**
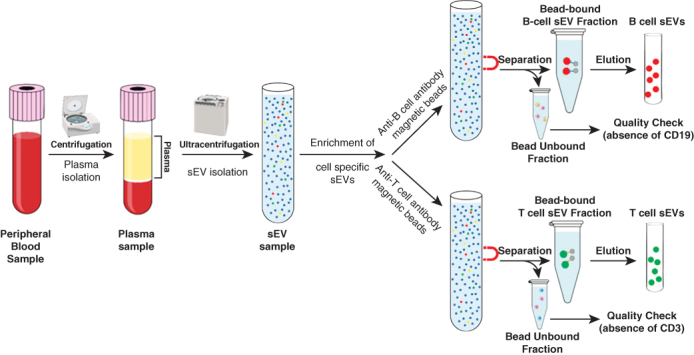
B-cell small extracellular vesicle enrichment as biomarkers of antibody mediated rejection.

**Central Message**

There is a critical need for biomarkers of AMR. We developed a novel circulating B-cell extracellular vesicle platform as biomarker of AMR in lung transplantation.

Lifelong surveillance for allograft rejection is a major cornerstone of care for patients after lung transplant. Antibody-mediated rejection (AMR) significantly contributes to allograft dysfunction, yet its noninvasive diagnosis and treatment monitoring remain challenging.^1^ We have been investigating cell/tissue-specific small extracellular vesicles (sEVs) as noninvasive biomarkers for transplant rejection monitoring, including in lung transplantation.^2^^,^^3^ We recently developed a novel biomarker platform for acute cellular rejection (ACR) monitoring based on T-cell sEV cargo profiling.^4^ Building on this, we hypothesized that because ACR leads to changes in circulating T-cell sEVs, AMR would induce changes in circulating B-cell sEV cargoes, enabling its noninvasive diagnosis. B-cell–specific EV enrichment from peripheral blood has never been reported before and the biology of circulating B-cell EVs in transplantation remains largely unexplored. Thus, our platform introduces a novel approach to noninvasive AMR monitoring. Herein, we report the development of a B-cell–specific sEV platform that demonstrated distinct cargo changes in a patients undergoing lung transplant with AMR compared with 2 patients without AMR/ACR.

We studied 3 bilateral lung transplant recipients (patients 1-3). Patient 1 developed mixed AMR and ACR; patients 2 and 3 did not have ACR or AMR. Peripheral blood was collected on postoperative days (PODs) 1, 3, and 30, with surveillance transbronchial lung biopsies performed on POD 30. Unlike patients 2 and 3, patient 1 developed de novo donor-specific antibodies on POD 30 and presented with dyspnea and pulmonary infiltration on computed tomography (Figure 1, *A* and *B*). Pulmonary function tests were performed for functional vital capacity and forced expiratory volume in 1 second measurements (Figure 1, *C*). This demonstrated improvement in functional vital capacity and forced expiratory volume in 1 second compared with pretransplant measurements in all 3 patients, but the least improvement was seen in patient 1 (Figure 1, *C*). This patient received treatment for mixed ACR/AMR with thymoglobulin, high-dose steroids, carfilzomib, tocilizumab, and intravenous immunoglobulin therapy. Patients 2 and 3 had uneventful postoperative courses without development of donor-specific antibodies and biopsies demonstrating no ACR or AMR.

**Figure 1:**
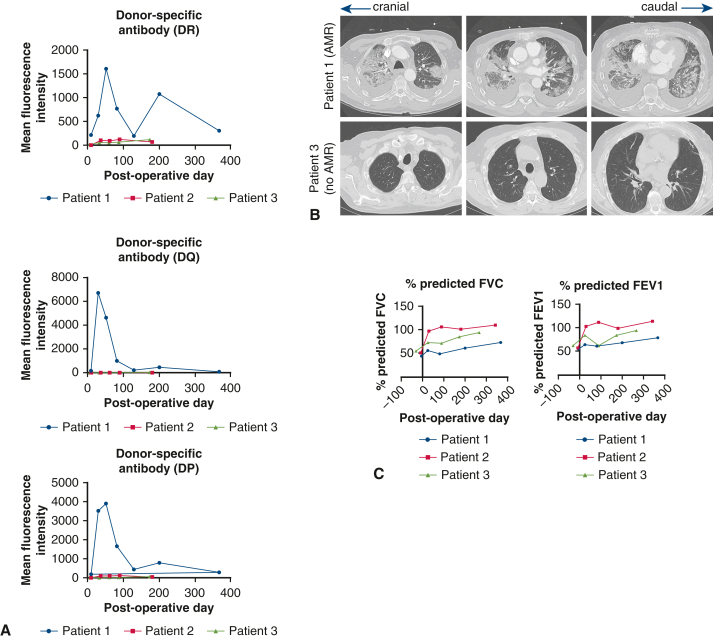
Pertinent clinical data for patients 1 to 3 are shown. A, Donor-specific antibody titers for human leukocyte antigen (*HLA*) DQ, HLA-DP, and HLA-DR in patients 1 through 3 are shown. Patient 1 showed acute upregulation of donor-specific antibodies at the postoperative day 30 time point. B, Computed tomography imaging is shown for patients 1 and 3. Acute bilateral infiltration was noted in patient 1 at the postoperative day 30 time point. C, Pulmonary function tests for patients 1 through 3 are shown. Compared with pretransplant lung volumes, all 3 patients demonstrated improved forced vital capacity (*FVC*) and forced expiratory volume in 1 second (*FEV1*) after bilateral lung transplantation, but patient 1 showed the least improvement.

We isolated plasma sEVs enriched with exosomes using serial ultracentrifugation (Appendix E1), characterized sEVs per the Society of Extracellular Vesicles guidelines,^5^ including expression of exosome markers CD63, flotillin-1, TSG101, alix-1, and CD81 on Western blot, along with absence of calnexin (endoplasmic reticulum marker), cytochrome c (mitochondria marker), and apolipoprotein E (plasma lipoprotein marker) (Figure E1, *A*, and Table E1). By nanoparticle tracking analysis, the isolated nanoparticles phenotyped as sEVs (Figure E1, *B*-*E*). B-cell–specific sEVs were enriched from the plasma sEV pool using anti-CD19 antibody-conjugated beads (Figure 2, *A*). As quality control, the bead-unbound sEVs were analyzed for the absence of CD19 but presence of flotillin-1 (Figure 2, *B*). The bead-bound sEVs representing B-cell sEVs were analyzed by nanoparticle tracking analysis (Figure 2, *C*) and Western blot for expression of B-cell activation protein markers, including CD20, class II human leukocyte antigen (HLA)-DR, CD38, interferon gamma (IFNγ), interleukin 6 receptor, and B-cell activating factor receptor (Figure 2, *D*). Furthermore, several of these markers were more highly expressed at the POD 30 time point of AMR diagnosis in patient 1. Similarly, we enriched T-cell sEVs using anti-CD3 antibody-conjugated beads and analyzed both bound and unbound fractions (Figure 2, *E*-*G*).^4^ Collectively, this suggested that AMR may lead to changes in protein cargoes of circulating B-cell sEVs.

**Figure 2:**
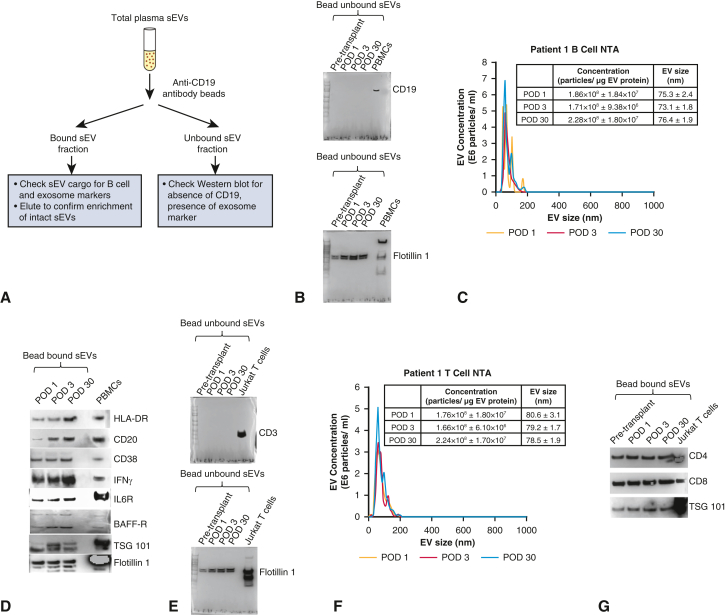

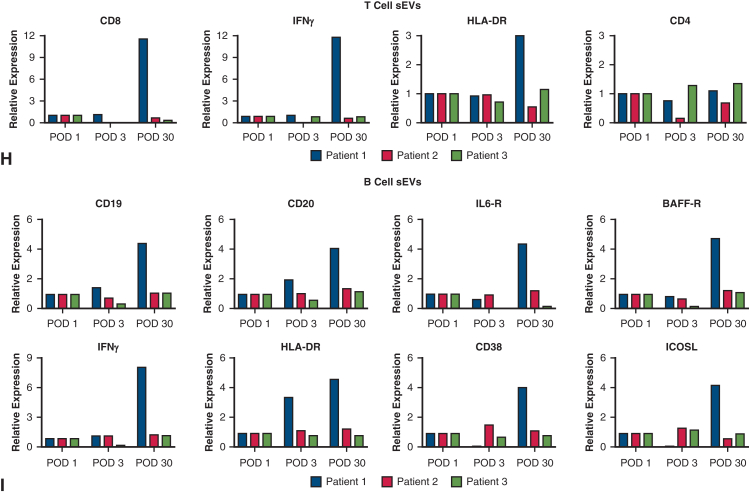
Circulating T-cell sEV and B-cell small extracellular vesicles (*sEV*) cargoes in a patient undergoing lung transplant with mixed acute cellular rejection/antibody-mediated rejection (*ACR/AMR*). A, Workflow for B cell sEV enrichment and downstream analysis is shown. B, Anti-CD19 antibody bead-unbound sEVs were analyzed by Western blot for CD19 absence and flotillin-1 (exosome marker) expression. Patient 1 data are shown. Peripheral blood mononuclear cells (*PBMCs*) are positive control. C, For patient 1, nanoparticle tracking analysis (*NTA*) of eluted anti-CD19 antibody bead-bound sEVs is shown. D, For patient 1, Western blot for expression of B-cell activation markers human leukocyte antigen (*HLA*) DR, CD20, CD38, interferon gamma (*IFNγ*), interleukin 6 receptor (*IL6-R*), B-cell activating factor receptor (*BAFF-R*), and exosome markers TSG101 and flotillin-1 in B-cell sEVs is shown. E, Western blot of T-cell sEVs, enriched from plasma total sEV pool using anti-CD3 antibody beads, is shown for CD3 absence and flotillin-1 expression in patient 1. Jurkat T-cells positive control is shown. F, NTA of eluted T-cell sEVs is shown for patient 1. G, Western blot for expression of T-cell markers, CD4, and CD8 in T-cell sEVs is shown for patient 1. H, Reverse transcription quantitative real-time polymerase chain reaction (*RT-qPCR*) assays for T-cell sEV messenger RNA cargoes CD8, IFNγ, HLA-DR, and CD4 in patients 1 through 3 at postoperative days (*PODs*) 1, 3, and 30 time points are shown. Upregulation of T-cell activation markers CD8, IFNγ, and HLA-DR, was seen at POD 30 time point in patient 1 only. I, RT-qPCR assays for B-cell sEV messenger RNA cargoes, CD19, CD20, IL6-R, BAFF-R, IFNγ, HLA-DR, CD38, and inducible co-stimulator ligand (*ICOSL*), in patients 1 through 3 at PODs 1, 3, and 30 time points are shown. Upregulation of these markers was observed in patient 1, POD 30 time point of AMR.

Next, we developed methodologies for characterization of messenger RNA (mRNA) cargoes of sEVs by reverse transcription quantitative real-time polymerase chain reaction.^E1^ For ACR, we quantified T-cell sEV mRNA cargoes for markers of T-cell alloreactivity (CD8, IFNγ, HLA-DR, and CD4) (Table E2). We compared relative expression at PODs 3 and 30 to POD 1 (baseline). Patient 1 with mixed ACR/AMR showed upregulation of markers of T-cell alloreactivity, including CD8, IFNγ, and HLA-DR on POD 30 (Figure 2, *H*), whereas patients 2 and 3 exhibited marker expression levels similar to baseline across all time points (Figure 2, *H*). During AMR, alloreactive B-cells become activated and upregulate expression of several markers, including CD19, CD20, interleukin 6 receptor, B-cell activating factor receptor, IFNγ, HLA-DR, CD38, and inducible co-stimulator ligand.^E2^ Therefore, we assessed whether circulating B-cell sEVs carry the mRNA signatures for these markers, and if so, whether they are upregulated with AMR. We developed reverse transcription quantitative real-time polymerase chain reaction assays for these 8 mRNAs for analysis in B-cell sEVs. In patient 1, these markers of B-cell activation were upregulated at the POD 30 time point compared with the PODs 1 and 3 time points, coinciding with AMR/ACR diagnosis and treatment (Figure 2, *I*). Patients 2 and 3 showed unchanged B-cell sEV marker expression across all time points (Figure 2, *I*). These results demonstrate that B-cell sEV cargoes, which reflect increased B-cell alloreactivity, were selectively upregulated only in the patient who developed AMR.

## Conclusions

We detail the development of a circulating B-cell sEV platform that demonstrated time-specific, distinct cargo changes in a patient undergoing lung transplant diagnosed with mixed ACR/AMR compared with a matched analysis in 2 patients undergoing lung transplant without rejection. This case series introduces and supports further investigation of this platform. If validated, the B-cell sEV platform would constitute a novel noninvasive biomarker for AMR surveillance in lung transplantation, supplementing/minimizing the need for invasive, resource-intensive transbronchial biopsy and its associated complications. It would permit more frequent AMR monitoring via simple peripheral blood sampling, thus directly influencing long-term care of patients undergoing lung transplant.

## Conflict of Interest Statement

The authors reported no conflicts of interest.

The *Journal* policy requires editors and reviewers to disclose conflicts of interest and to decline handling or reviewing manuscripts for which they may have a conflict of interest. The editors and reviewers of this article have no conflicts of interest.

## Figures and Tables

**Figure E1:**
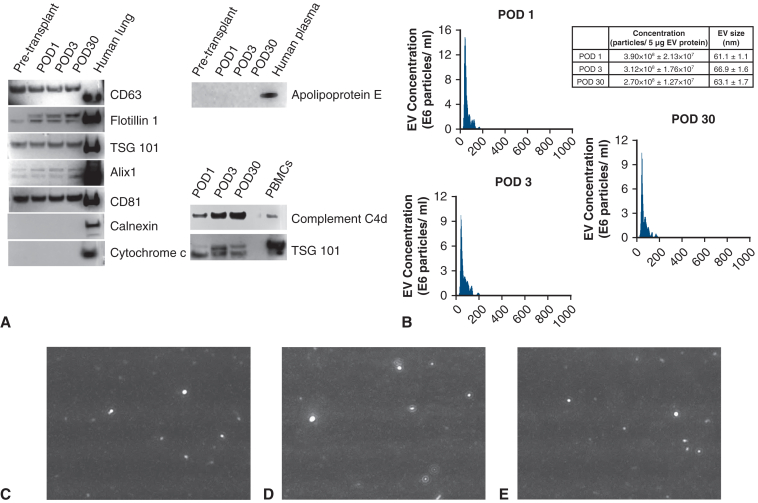
Characterization of plasma small extracellular vesicles (*sEVs*) in patient 1 is shown. A, Western blot analysis of plasma sEVs for exosome markers is shown: CD63, flotillin-1, TSG 101, alix-1, and CD81. To assess the plasma sEV isolates for absence of contamination from cellular organelles, Western blot for calnexin (endoplasmic reticulum marker) and cytochrome c (mitochondrial marker) was performed. Human lung tissue protein extract is shown as positive control. To assess for absence of contamination in the sEV isolates from blood lipoproteins, Western blot for apolipoprotein E was performed. Human plasma is shown as positive control. By Western blot, patient 1 with mixed acute cellular rejection + antibody mediated rejection (ACR + AMR) also showed increased expression of complement C4d in the sEV isolate from postoperative day (*POD*) 30 compared with POD 1 and 3 time points. Peripheral blood mononuclear cells (*PBMC*) protein isolate is shown as positive control. TSG 101 is an exosome marker. B, Nanoparticle tracking analysis (*NTA*) of plasma sEVs for PODs 1, 3, and 30 time points in patient 1 are shown. This demonstrated that the phenotype of the majority of the isolated sEVs were in the exosome size range, <150 nm. C, D, and E, Representative NTA videos of plasma sEVs for PODs 1 (C), 3 (D), and 30 (E) in patient 1 are shown.

**Table E1:** Antibodies utilized for Western blot analysis and for antibody bead conjugation

Antibody	Biotech company	Catalogue no.	Dilution
CD3-ε (UCH-T1) Mouse monoclonal	Santa Cruz Biotechnology	SC-1179	Antibody-conjugated beads
CD19 (SJ25-C1) Mouse monoclonal	Santa Cruz Biotechnology	SC-18896	Antibody-conjugated beads
Human leukocyte antigen DR (LN-3) Mouse monoclonal	Novus Biologicals Inc	NBP2-45310	1:1000
CD20 (AT80) Mouse monoclonal	Santa Cruz Biotechnology	Sc-58982	1:500
CD38 (H11) Mouse monoclonal	Santa Cruz Biotechnology	Sc-374650	1:500
Interferon gamma Rabbit polyclonal	Proteintech	15365-1-AP	1:1000
Interleukin 6 receptor Mouse monoclonal	Proteintech	66855-1-Ig	1:1000
B-cell activating factor receptor (F-3) Mouse monoclonal	Santa Cruz Biotechnology	SC-365610	1:500
Tumor suppressor gene 101 Mouse monoclonal	Proteintech	67381-1-Ig	1:1000
Flotillin 1 Mouse monoclonal	Proteintech	67968-1-Ig	1:1000
CD4 (MT310) Mouse monoclonal	Santa Cruz Biotechnology	Sc-19641	1:500
CD8 (UCH-T4) Mouse monoclonal	Santa Cruz Biotechnology	Sc-1181	1:500

**Table E2:** Primers utilized for reverse transcription qualitative polymerase chain reaction of messenger RNA cargoes of T-cell small extracellular vesicles and B-cell small extracellular vesicles

Gene	Primer sequences
CD8	Forward: 5′- CCCACTTTGTAGCCCCATCAC-3′
	Reverse: 5′- TCAGTCCTCCAGCACACTCTG-3′
Interferon gamma	Forward: 5′- TTTTGAAGAATTGGAAAGAGGA-3′
	Reverse: 5′- CACTTGGATGAGTTCATGTATTG-3′
Human leukocyte antigen-DR	Forward: 5′- TGGGACCATCTTCATCATCAAGG--3′
	Reverse: 5′- GGGCATTCCAT AGCAGAGACAGAC-3′
CD4	Forward: 5′- TCCTTCCCACTCGCCTTTACA-3′
	Reverse: 5′- AGCACCCACACCGCCTTCTCC-3′
CD19	Forward: 5′- TGCCCCGTCTTATGGAAACC-3′
	Reverse: 5′- CTCTTCTTCTGGGCCCACTC-3′
CD20	Forward: 5′- CTC TCT GGG GAG GCA TTA TGT A -3′
	Reverse: 5′- GTA ACA GTA TTG GGT AGA TGG GGA G -3′
Interleukin 6 receptor	Forward; 5′- TGG TGG ATG TTC CCC CCG AG -3′
	Reverse: 5′- TCC TGG GAA TAC TGG CAC GG -3′
B-cell activating factor receptor	Forward: 5′- GTG GGT CTG GTG AGC TGG AG -3′
	Reverse: 5′- GGT GGT TCC TGG TCT TCC C -3′
CD38	Forward: 5′-TGCTGATGACCTCACATGGT-3′
	Reverse: 5′- CCATTGAGCATCACATGGAC-3′
Inducible co-stimulator ligand	Forward: 5′- TGG AAG AGGTGG TCA GGC -3′
	Reverse: 5′- TTA GGC TAT TGT CCGTTG-3′

## Appendix E1 Supplemental Methods

### Antibodies

Antibodies were purchased and recommended dilutions in the provided data sheets for Western blot analysis (Table E1) were followed. Monoclonal anti-CD3 (SC-1179), and anti-CD19 (SC-18896) antibodies (Santa Cruz Biotechnologies Inc) were used to conjugate with the N-hydroxysuccinamide magnetic beads (88827) from Pierce Inc. Horseradish peroxidase-conjugated secondary anti-rabbit and anti-mouse antibodies for Western blot analysis were purchased from Santa Cruz Biotechnologies Inc.

### Small Extracellular Vesicles Isolation From Plasma and Characterization

The Institutional Review Board or equivalent ethics committee of Washington University, St Louis (No. 201705135; 2022) approved the study protocol and publication of data. The patients provided informed written consent for the publication of the study data. Patients’ blood samples were collected on postoperative days (PODs) 1, 3, and 30 along with the patient clinical and transbronchial lung allograft biopsy information. Peripheral blood samples (5 mL) were collected in an EDTA tube and centrifuged at 2000 × g for 20 minutes. The supernatant representing the plasma was collected and frozen at −80 °C. Samples were then shipped to Yale University for small extracellular vesicles (sEVs) isolation and downstream analysis. Isolation and characterization of sEVs was performed with modification of previously published methodologies from our laboratory.^2^, ^3^, ^4^ Because our group has transitioned to potential translation of the sEV methodologies to the clinical setting, the isolation protocol was modified to performing ultracentrifugation as the primary modality, excluding size-exclusion chromatography techniques, which are very time consuming and resource intensive, and more difficult to achieve wide application. Our goal was to simplify the sEV isolation technique without compromising quality control so that the technique can be more easily and widely adopted. In making this change, we performed extensive quality testing to ensure that the sEV cargo analysis experiments were not compromised.

Plasma sample ultracentrifugation methodologies were adopted to isolate sEVs by spinning the plasma at 130 000 × g twice for 2 hours and the sEV pellet was resuspended in phosphate buffered saline. Protein concentration was obtained using absorbance spectrometry and 5 μg sEV protein equivalent was run by nanoparticle tracking analysis (NS300; Malvern Instruments) to obtain nanoparticle size distribution and concentration. The mean particle size distribution was <100 nm, and in any preparations that the mean size was >100 nm, the sample was not utilized for further downstream sEV applications. To confirm enrichment of exosomes in the sEV preparation, Western blot was performed to assess for expression of exosome markers, CD63, CD81, TSG101, flotillin-1, and alix-1. To make sure that the sEV preparation was not contaminated with intracellular organelles, Western blot was performed to check for absence of calnexin (endoplasmic reticulum marker) and cytochrome c (mitochondria marker), along with appropriate positive control. Because the size of apolipoproteins in plasma can be in the same size range as sEVs, Western blot for absence of apolipoprotein in the sEV preparation was also performed along with total human plasma protein as positive control.

### Enrichment of T-cell and B-cell sEVs Using Affinity Antibody Coupled Methodology

Specific antibodies for T-cells (anti-CD3) and B-cells (anti-CD19) were covalently conjugated to N-hydroxysuccinamide magnetic beads per manufacturer's protocol. sEVs (1.5-2.0 × 10^10^ particles based on sample availability) were incubated with antibody-magnetic beads complex overnight at 4 °C. Using the magnetic stand, bead-bound sEVs and unbound EVs were separated. The bound fraction representing the T-cell EV subsets, or B-cell subsets were washed with phosphate buffered saline and used for the downstream applications (RNA and protein cargo). As quality control, the bound sEV fraction was assessed by Western blot for expression of cell-specific markers (T-cell or B-cell based on the antibody used) and the unbound sEV fraction was assessed for absence of cell-specific markers and presence of exosome marker, flotillin-1, along with appropriate positive control.

### sEVs RNA and Protein Cargo Analysis

Total RNA was extracted from T-cell specific and B-cell specific sEVs by using TRIzol reagent (Invitrogen) followed by phenol chloroform extraction methodology.^4^ RNA quality and concentration was measured on Nanodrop One (Thermo Fisher). Bound sEVs were lysed using radioimmunoprecipitation assay buffer and separated on NuPAGE 4% to 12% Bis-Tris gel and transferred onto iBlot 2 nitrocellulose membrane (Invitrogen). The blot was blocked with 5% milk, incubated with specific primary antibody, followed by horseradish peroxidase-conjugated secondary antibody. Target protein expression was detected by chemiluminescence using Image quant LAS Amersham ImageQuant 800 (Cytivia Lifesciences).^4^

### Messenger RNA Expression Analysis Using Reverse Transcription Quantitative Real-Time Polymerase Chain Reaction

RNA enriched from anti-CD3 bound sEVs or from anti-CD19 bound sEVs was used for amplification of complementary DNA (cDNA) using Vilo cDNA reverse transcription kit (11754050) (Thermo Fisher Scientific). Reverse transcription quantitative real-time polymerase chain reaction reactions were performed using synthesized cDNA along with forward and reverse primers (Table E2) mixed with PowerUp SYBR Green master mix (Thermo Fisher Scientific) in each reaction. The Reverse transcription quantitative real-time polymerase chain reaction reactions were conducted on the QuantStudio 3 Real-Time qPCR system (Thermo Fisher Scientific). For each messenger RNA candidate biomarker, expression levels were normalized to internal control b-actin messenger RNA expression. Obtained relative quantitative values from cycle threshold were normalized to β-actin expression for that sample and quantified using the 2^−ΔΔ*C*^_T_ method.^E2^
